# Active Learning-Guided Hit Optimization for the Leucine-Rich Repeat Kinase 2 WDR Domain Based on In Silico Ligand-Binding Affinities

**DOI:** 10.1021/acs.jcim.5c00588

**Published:** 2025-05-26

**Authors:** Filipp Gusev, Evgeny Gutkin, Francesco Gentile, Fuqiang Ban, S. Benjamin Koby, Fengling Li, Irene Chau, Suzanne Ackloo, Cheryl H. Arrowsmith, Albina Bolotokova, Pegah Ghiabi, Elisa Gibson, Levon Halabelian, Scott Houliston, Rachel J. Harding, Ashley Hutchinson, Peter Loppnau, Sumera Perveen, Almagul Seitova, Hong Zeng, Matthieu Schapira, Artem Cherkasov, Olexandr Isayev, Maria G. Kurnikova

**Affiliations:** † Department of Chemistry, Mellon College of Science, 6612 Carnegie Mellon University, Pittsburgh, Pennsylvania 15213, United States; ‡ Computational Biology Department, School of Computer Science, Carnegie Mellon University, Pittsburgh, Pennsylvania 15213, United States; § Department of Chemistry and Biomolecular Sciences, 12365 University of Ottawa, Ottawa, Ontario K1N 6N5, Canada; ∥ Ottawa Institute of Systems Biology, Ottawa, Ontario K1H 8M5, Canada; ⊥ Vancouver Prostate Centre, 8166 The University of British Columbia, Vancouver, British Columbia V6H 3Z6, Canada; # Structural Genomics Consortium, 7938 University of Toronto, Toronto, Ontario M5G 1L7, Canada; ∇ Princess Margaret Cancer Centre, 7989 University Health Network, Toronto, Ontario M5G 2M9, Canada; ○ Department of Pharmacology & Toxicology, University of Toronto, Toronto M5G 2C8, Canada

## Abstract

The leucine-rich repeat kinase 2 (LRRK2) is the most mutated gene in familial Parkinson’s disease, and its mutations lead to pathogenic hallmarks of the disease. The LRRK2 WDR domain is an understudied drug target for Parkinson’s disease, with no known inhibitors prior to the first phase of the Critical Assessment of Computational Hit-Finding Experiments (CACHE) Challenge. A unique advantage of the CACHE Challenge is that the predicted molecules are experimentally validated in-house. Here, we report the design and experimental confirmation of LRRK2 WDR inhibitor molecules. We used an active learning (AL) machine learning (ML) workflow based on optimized free-energy molecular dynamics (MD) simulations utilizing the thermodynamic integration (TI) framework to expand a chemical series around two of our previously confirmed hit molecules. We identified 8 experimentally verified novel inhibitors out of 35 experimentally tested (23% hit rate). These results demonstrate the efficacy of our free-energy-based active learning workflow to explore large chemical spaces quickly and efficiently while minimizing the number and length of expensive simulations. This workflow is widely applicable to screening any chemical space for small-molecule analogs with increased affinity, subject to the general constraints of RBFE calculations. The mean absolute error of the TI MD calculations was 2.69 kcal/mol, with respect to the measured *K*_D_ of hit compounds.

## Graphical abstract

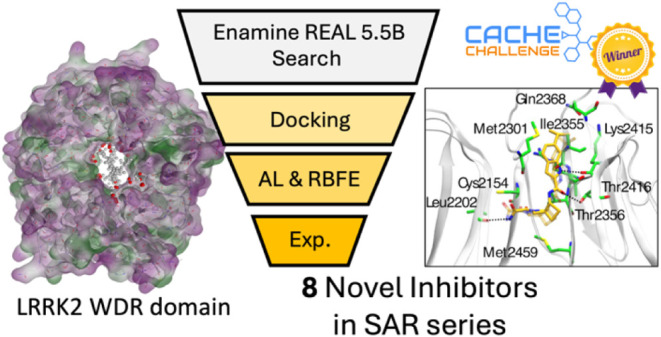

## 1 Introduction

The Critical Assessment of Computational Hit-Finding Experiments (CACHE) Challenge^1^ is a series of scientific competitions that benchmark computational approaches to identify small molecules capable of binding to specific molecular targets with the goal of stimulating *in silico* drug discovery for rare and understudied medicinally important targets. The objective of CACHE Challenge #1 was to find small-molecule binders to the WDR domain of the leucine-rich repeat kinase 2 (LRRK2), a multidomain protein and a Parkinson’s disease (PD) target. LRRK2 is the most mutated protein in familial PD, and its mutation leads to pathogenic hallmarks of the disease.^2–4^ While inhibitors and PROTACs targeting the kinase domain of LRRK2 have been reported,^5–7^ no ligand so far targets the juxtaposed WDR domain of the protein,^8^ even though WDR domains have proven druggable in other proteins.^9^ A recurrent pathogenic mutation maps at the interface of the LRRK2 WDR dimer, highlighting the disease relevance of this domain.^8^ CACHE Challenge #1 included two rounds of prediction and experimental confirmation, allowing participants to incorporate insights gained from the first round into their predictions for the second round. The first phase focused on hit identification by *in silico* screening of commercially available libraries^10,11^ followed by experimental testing, yielding five confirmed hits from our selection.^12^

Here we report our winning submission on the optimization study of two LRRK2 WDR domain binders. These ligands, Hit 1 and Hit 2 (Figure 1), had the highest experimental binding affinities among five initially validated hits from “round 1”. The computational pipeline included the selection from a commercial library of ligands sharing a common substructure with our hits, followed by docking and optimized molecular dynamics (MD) thermodynamic integration (TI) simulations,^13^ guided by active learning (AL),^14^ to compute the RBFE of compounds to the target. We screened ∼5.5B commercially available compounds, selected ∼25 000 ligands for AL-RBFE calculations, and computed RBFEs for 672 ligands. Based on the computed RBFEs, 75 molecules were selected for experimental validation, and 35 were further tested experimentally. Binding of 8 ligands to the WDR domain was confirmed by surface plasmon resonance (SPR) and ^19^F-nuclear magnetic resonance (NMR) for fluorinated molecules.

**Figure 1:**
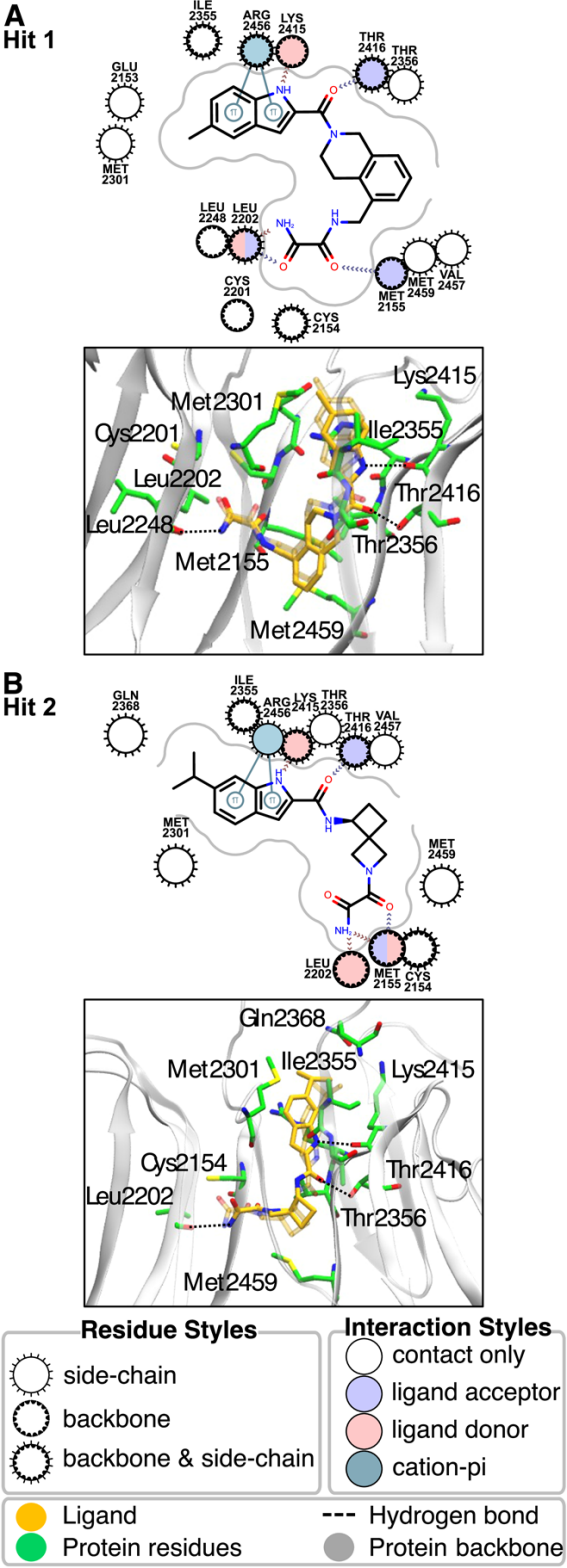
Experimentally confirmed hits identified in the first phase of the CACHE Challenge #1 selected for subsequent optimization.

These findings pave the way for the next steps toward the discovery of high-affinity selective compounds targeting the WDR40 domain of LRRK2. Our compound selection received the highest score from an independent committee of biophysics and medicinal chemistry industry experts in the CACHE Challenge #1 competition.^15^

## 2 Results and Discussion

### 2.1 Computational Pipeline

We developed a comprehensive pipeline for hit optimization, leveraging our active learning (AL) workflow for relative binding free energy (RBFE) calculations^14^ (see Methods section 4). The Enamine REAL database^10^ was used for virtual screening, which comprised 5.5 billion small-molecule compounds at the start of this work. The first step of the virtual screening protocol was to filter this set using two distinct SMARTS patterns for each hit (Figure 2B): the first pattern contained Murcko scaffolds and the oxamide group, and the second pattern comprised solely of Murcko scaffolds derived from the initial hits. This resulted in two sets of molecules: i) a set of the closest analogs and ii) a set of more distant analogs, further termed “general analogs”. For the closest analogs (250 molecules), we conducted template docking to the MD representative structures of the protein–ligand complexes associated with Hit 1 and Hit 2. Thus, 214 docked molecules (46 analogs of Hit 1 and 168 analogs of Hit 2) were selected for RBFE MD simulations. To improve diversity within the closest analogs of Hit 1, we further performed a nearest neighbor (NN) search among general analogs to identify molecules with high similarities to the top 9 molecules exhibiting the lowest computed RBFEs. These 27 identified molecules were subsequently incorporated into the set of the closest analogs (see Section 4.1.1.2 for details), with docking and RBFE calculations performed in an analogous manner to the initial batch. The RBFEs were converted to ABFEs (see the Methods section 4 for details). This set is further termed the pre-AL set.

**Figure 2:**
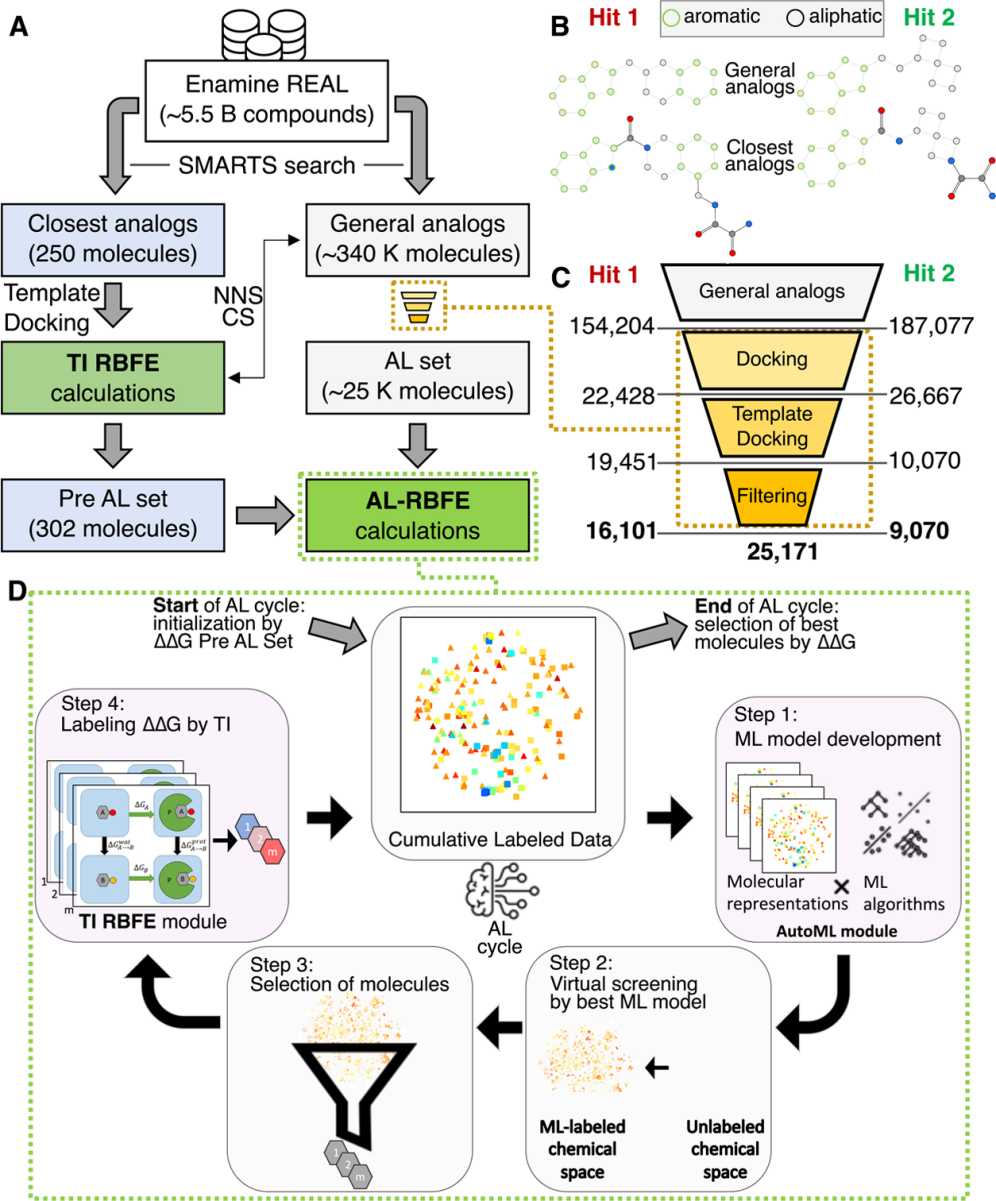
Overview of computational approach for hit optimization. (A) General scheme of computational pipeline used for optimization of both hits (see main text for description). The blocks corresponding to closest analogs, general analogs, and RBFE calculations are shown in blue, gray, and green, respectively. NNS stands for the nearest neighbors search, and CS stands for curated selection (see the Methods section 4 for details). (B) SMARTS patterns of the closest analogs and the general analogs used for Hit 1 and Hit 2. (C) Virtual screening of the general analogs of Hit 1 and Hit 2. The numbers of molecules for Hit 1 and Hit 2 after each step of the pipeline are shown. (D) General scheme of the automated computational workflow for RBFE calculations guided by AL (AL-RBFE). The workflow includes two main modules: AutoML and MD TI RBFE and the four principal steps. The chemical space is shown as 2D t-SNE plots. analogs of Hit 1 and Hit 2 with computed ΔΔ*G* are depicted as colored squares and triangles consistent with the color scheme on Figure 4.

For general analogs (∼340,000 molecules), we first performed docking without a template and filtered by docking score. Subsequently, we performed docking with a template using the same protocol as we employed for the closest analogs. We next conducted additional filtering of the docked ligands based on docking score and RMSD with respect to the template. The resulting set comprised approximately ∼16,000 analogs for Hit 1 and ∼9,000 analogs for Hit 2. This set (∼25,000 molecules) is referred to as the AL set (see Figure 2).

The theoretical background of AL as well as a detailed overview of the AL-RBFE workflow are provided in our previous work.^14^ Briefly, the AL-RBFE workflow is an iterative procedure in which, at each iteration, molecules with computed MD TI RBFE are used to train an ML model that predicts the RBFE of a ligand based on its chemical structure. After the ML model is trained, it predicts the RBFE for all the molecules from the data set and uses these predictions to select molecules for the next round of RBFE calculations with MD TI simulations. In this work, we used ABFE instead of RBFE to allow for screening analogs of both hits with the same ML model. Importantly, we still performed MD TI simulations to compute RBFEs, not ABFEs, after which we converted RBFEs to ABFEs (see Methods section 4 for details) and then used these data for training the ML model.

### 2.2 Perturbation Map for Relative Binding Free Energy Calculations

The perturbation map for MD TI RBFE calculations for the Hit 1 analogs is presented in Figure S1. For all Hit 1 analogs from the pre-AL set, RBFE calculations were performed using Hit 1 as a reference ligand. In contrast, the RBFE calculations of the general analogs were performed using ligand X as a reference ligand. Ligand X was derived from the Hit 1 analogue A, which had the lowest ABFE among the molecules from the pre-AL set (see Figure S1). Ligand X was utilized as a reference ligand for the general analogs due to the difficulties in preparing initial structures for RBFE calculations. Specifically, when trying to compute RBFEs for some molecules from the AL-1 set, we found that the initial conformations of these substituents were significantly distorted in the MD TI input structures. The reason for this distortion was that substituents at the 5 position of the 1,2,3,4-tetrahydroisoquinoline were forced to align with the methyloxamide group of the reference ligand (Hit 1) when preparing the system for MD TI RBFE simulations (see Methods section 4). This artificial alignment may bias the sampling of this ligand and provide unreliable RBFEs. Moreover, in some cases, this led to the appearance of clashes between the ligand and the protein, with subsequent failures in MD simulations. To address this challenge, we created ligand X from ligand A by substituting a methyloxamide group with hydrogen. Using ligand X as a reference for RBFE calculations allowed the substituents of challenging ligands to preserve their initial conformations. At the same time, it also allowed for more optimal RBFE calculations for ligands that do not have substituents at position 5 of the 1,2,3,4-tetrahydroisoquinoline. While using Hit 1 as a reference ligand in these cases would require the complete annihilation of the methyloxamide group, but using ligand X as a reference avoids this process and can thus increase the convergence of calculations.

### 2.3 Results of Active Learning-Guided Relative Binding Free Energy Calculations

We performed eight iterations of the AL-RBFE workflow. The pre-AL set was used as a training set to build the initial ligand ABFE-predicting ML model in the first iteration. For the next seven AL iterations (AL-1–AL-7), molecules for RBFE calculations were selected by an ML model (see Table S2 for the number of compounds selected at each AL iteration). Since Hit 1 had a higher binding affinity than Hit 2, iterations AL-1–AL-6 were performed only for the analogs of Hit 1. The last iteration AL-7 included both Hit 1 and Hit 2 analogs, with the aim of enriching predicted hits with analogs of Hit 2. After each round of MD TI simulations, computed RBFEs were converted to ABFEs (see Methods section 4 for details).

Results of all iterations of the AL-RBFE workflow are presented in Figure 3 and Table S2. The ABFEs were computed for 674 molecules in total (493 and 181 analogs of Hit 1 and Hit 2, respectively). Overall, we identified 102 analogs with computed ABFEs lower than the initial hits (87 and 15 analogs for Hit 1 and Hit 2, respectively). For Hit 1, ca. 80% of the analogs with improved ABFE were selected by the AL (70 of 87 molecules), with the rest identified from the pre-AL set. Improved analogs were identified at each AL iteration. The share of Hit 1 analogs with improved ABFE among all computed Hit 1 analogs was more than 1.5 times higher for the AL sets compared to the pre-AL sets: ca. 20% versus 13%, respectively. These results demonstrate the effectiveness of utilizing the AL to guide RBFE calculations.

**Figure 3:**
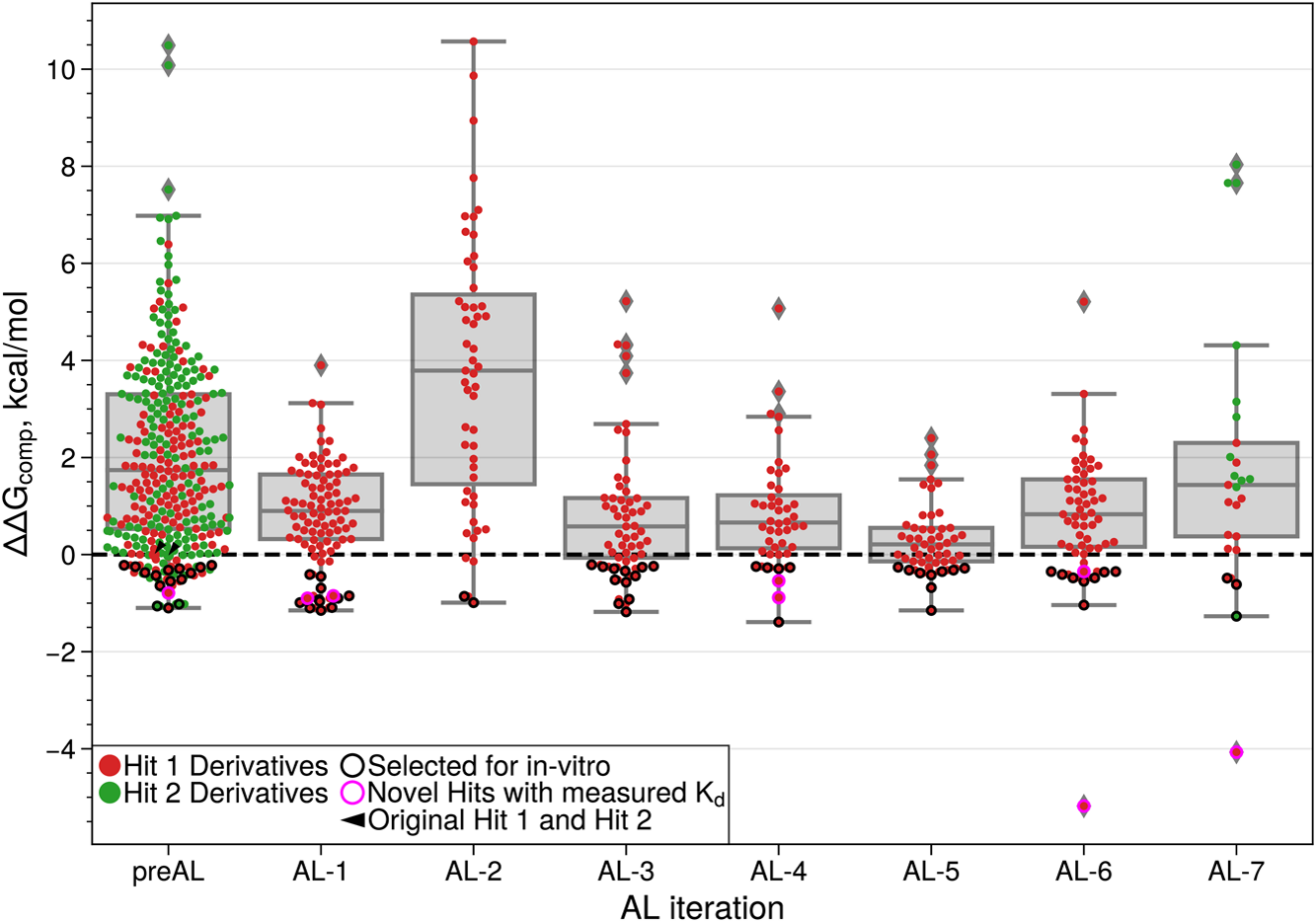
Computed MD TI RBFEs versus active learning iteration shown as a box plot. Hit 1 (red dots) and Hit 2 (green dots) analogs computed during the active learning cycle are shown in Figure 2D. The RBFEs of Hit 1 and Hit 2 are set to 0 kcal/mol and indicated by black arrows at the pre-AL step. The analogs of both hits selected for submission to the experimental evaluation are encircled in black or magenta colors. The magenta color shows the novel hits with measured *K*_D_ (Figure 5 and Table S3).

For more insight into the performance of the AL-RBFE workflow, we visualized the chemical space of all analogs (pre-AL and AL sets together) and molecules with computed ABFEs using t-SNE projections (Figure 4). The t-SNE plots were built for each individual AL iteration (Figure 4A) as well as for the results of all iterations together (Figure 4B). We can see that the computed molecules of the pre-AL set are distributed over different regions of chemical space instead of being localized in the same region, indicating a certain structural diversity among molecules of this set. The same trend is maintained during the rest of the iterations: AL selects molecules from different regions of the chemical space, from both the regions screened at the previous iterations and those unexplored at the preceding stages. Therefore, the use of AL to guide molecule selection increased the diversity of the ligands with an improved ABFE.

**Figure 4:**
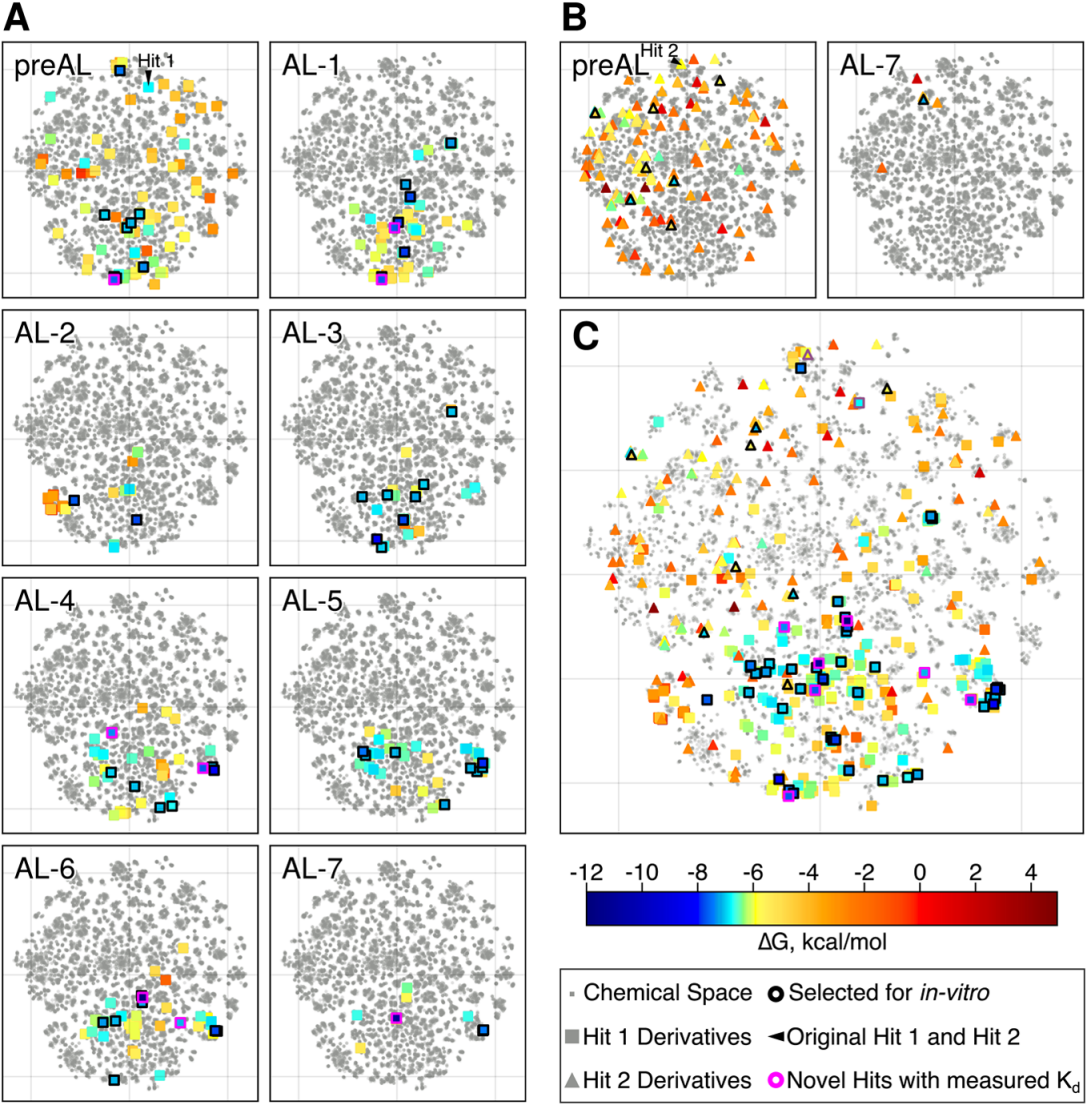
AL-guided calculated TI ABFEs shown as t-SNE projections of chemical space of Hit 1 and Hit 2 analogs. (A). t-SNE plots of each individual AL iteration for Hit 1 analogs. (B.) t-SNE plots of each individual AL iteration for Hit 2 analogs. (C). t-SNE plot of all iterations of active learning. Each molecule is shown as a point. Hit 1 and Hit 2 are indicated by black arrows. Molecules are colored by their computed ABFE, and the rest of molecules are shown in gray. The initial hits are circled by purple. Molecules selected for experimental in vitro validation are circled in black, and optimized hits confirmed experimentally are circled in magenta.

### 2.4 Experimentally Validated Hits

The selection of molecules for submission to the experimental assays was done according to the challenge budget (75 molecules or $10,000, whatever comes first) as follows: 70 molecules were greedily selected solely based on the most negative computed Δ*G* (67 derivatives of Hit 1 and 3 derivatives of Hit 2), and the remaining 5 molecules were selected across Hit 2 derivatives with negative Δ*G* yet chemically diverse representatives. The selected 75 molecules were quoted to the Enamine chemical vendor, 35 of which were procured and tested experimentally by surface plasmon resonance (SPR) at 50 μM (see Methods section 4, Experimental Methods section 4.5, and Surface plasmon resonance section 4.5.2). All Enamine-supplied compounds were >95% pure by HPLC analysis. Eleven hit candidates were advanced to dose–response experiments, eight of which had measurable dissociation constant *K*_D_ better than 250 μM and acceptable SPR sensorgrams (Chi^2^ < 10% *R*_max_; *T*(*K*_D_) > 1), with *K*_D_ values ranging from 18 to 230 μM (Figure 5 and Table S1). As some of the hits were fluorinated molecules, we used ^19^F-NMR as an orthogonal assay to confirm that binding was not assay-specific (Figure 5). We verified in a dynamic light scattering assay that compounds were soluble and did not aggregate at relevant concentrations (Figure 5 and Table S1).^16^ Therefore, the hit rate for the second round of the CACHE Challenge was 23%. CACHE typically discards SPR hits with less than 30% binding. While compound O1 displayed 27% binding, which is below this cutoff, and compound O2 did not display a binding signal from ^19^F-NMR, they belong to a chemical series with confirmed binding to LRRK2 and have acceptable quality descriptors (Chi^2^ < 10% *R*_max_ and *T*(*K*_D_) > 1). They were, therefore, included in the reported structure–activity relationships (SAR) (Figure 5).

**Figure 5:**
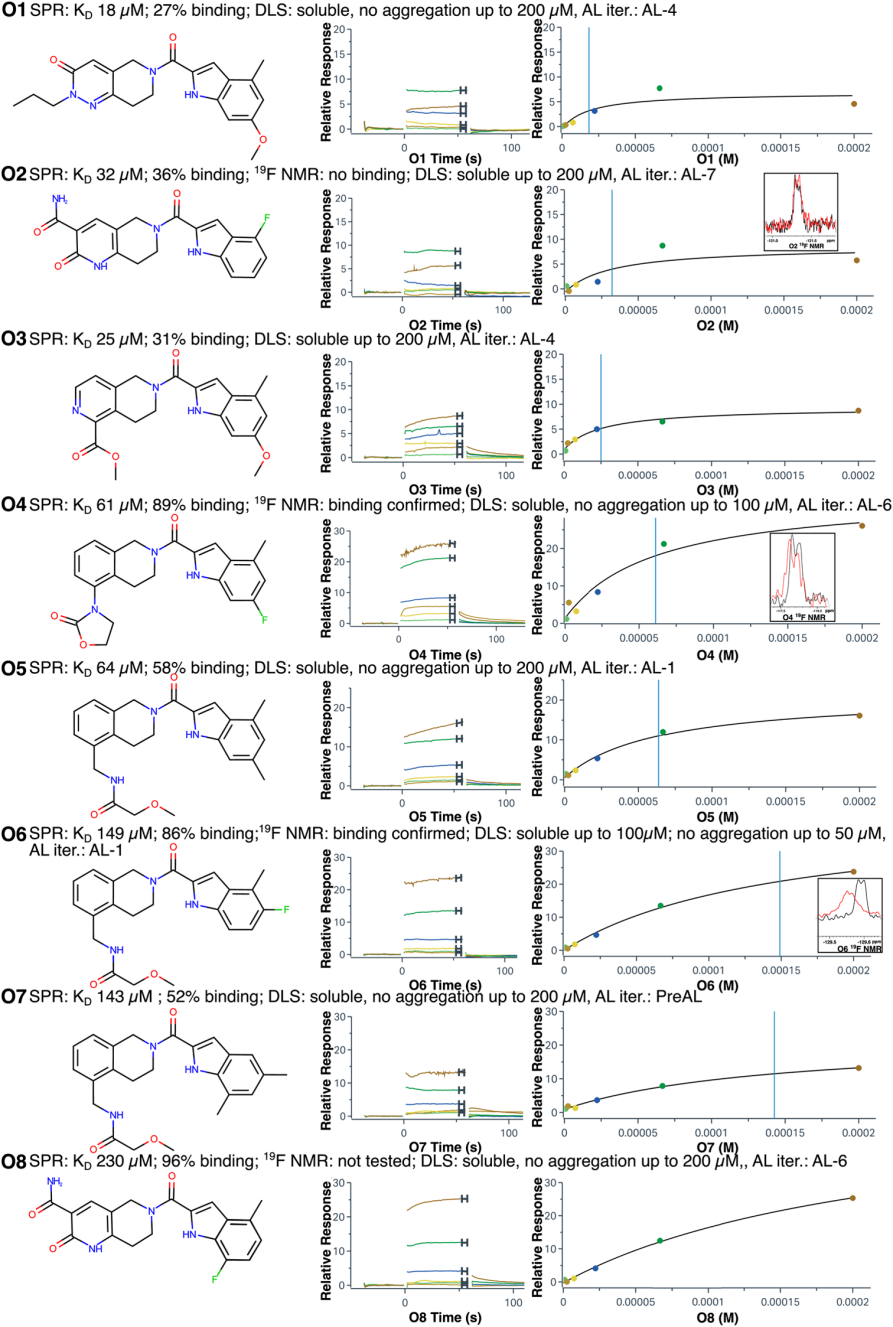
Experimentally measured binding properties of hit molecules. SPR sensorgrams, fragments (see Figure S2 for full spectra) of NMR spectra of fluorinated compounds (10 μM compound with 0 [black], 20 [red] μM protein), and chemical structures are shown. Solubility and aggregation of compounds as measured by DLS, as well as the set from which each compound was identified, are indicated.

The binding free energies of the O1–O8 hits are presented in Table S3. For two hits (O4, O6), binding was additionally confirmed by using ^19^F-nuclear magnetic resonance (NMR). The experimental dissociation constants (Table S3 and Table S1) ranged from 18 μM (though this compound showed only 27% binding by SPR) to 230 μM (the corresponding ABFE range is from −5.0 to −6.5 kcal/mol) with three stronger binders (*K*_D_ = 18–32 μM), two moderate binders (*K*_D_ = 61–64 μM), and four weak binders (*K*_D_ = 143–230 μM). The mean absolute error between computed and experimental ABFE for hit compounds was 2.69 kcal/mol. This demonstrates the ability of our proposed workflow to identify and expand a series with a reliable structure–activity relationship (SAR), even on a novel target without experimentally confirmed binding poses at the time of this work. Of the eight binders, only one was identified from the pre-AL set despite this set containing nearly half the simulated compounds, which suggests that active learning is more effective at selecting binders than naïve selection schemes; however, due to the small sample sizes involved, we are unable to make statistically significant conclusions regarding the two selection methods.

All optimized hits are analogs of Hit 1 and include an indole ring connected to the piperidine ring via a carbonyl bridge. Four hits (O4, O5, O6, and O7) also contain a benzene ring joined with the piperidine ring forming a 1,2,3,4-tetrahydroisoquinoline system. Three out of these five hits (O5, O6, and O7) have the same modification (the terminal amide of the oxamide group is substituted with the methoxymethyl group) and differ only by substituents in the indole ring. Hit O7 was from the pre-AL set, and two other hits (O6 and O5) were selected by AL at the first iteration (AL-1). Both computed and experimental ABFEs were within 0.6 kcal/mol, with hit O4 showing the strongest binding affinity among these hits. Compound O4 was different from the other hits: the methyloxamide was substituted with an oxazolidinone ring. This is the only optimized hit that contains three-ring systems. Despite having the highest computed ABFE, it showed a moderate experimental binding affinity with *K*_D_ of 61 μM.

In the other four compounds (O1, O2, O3, O8), the substituted benzene ring of 1,2,3,4-tetrahydroisoquinoline system is replaced with substituted six-membered heterocyclic aromatic cycles. Hits O8 and O2 contain 2-pyridone-carboxamide, compound O1 contains *N*-propylpyridazin-3-one, and Hit O3 contains a pyridine substituted with a methyl carboxylate group. Compounds O8 and O2 were selected at the last two iterations of the AL workflow and had ABFEs significantly lower compared to the rest of the analogs. These analogs differ from each other only in the substituents on the indole ring; however, while Hit O2 showed the third strongest binding affinity among all hits with a *K*_D_ of 32 μM, Hit O8 was a considerably weaker binder with a *K*_D_ of 230 μM. Notably, our MAE value is highly skewed by these two compounds. The exclusion of these compounds results in an MAE of 1.66 kcal/mol.

Hits O1 and O3, selected at the fourth AL iteration (the AL-4 set), had the same substituents in the indole ring but differed in the substituted ring system (*N*-propylpyridazin-3-one versus methylpyridine carboxylate). Despite the structural differences, both the computed and the experimental ABFE of these hits were relatively close to each other (less than a 0.4 kcal/mol difference). Hit O1 was the strongest binder among all hits with a *K*_D_ of 18 μM, and Hit O3 was the second strongest binder with a *K*_D_ of 25 μM. Importantly, all three strongest binders (O1, O2, O3) belonged to the set of general analogs of Hit 1 and had relatively diverse structures.

## 3 Conclusions

Recent advances in molecular modeling and machine learning technologies have paved the way for the development of novel computational approaches in biomedical applications. This paper further demonstrates the utility of the AL-RBFE combined approach^14^ for the identification and expansion of a series of small-molecule binders to a target protein. The approach combines accurate physics-based molecular simulations and ML methods, which were effective even for a challenging target with limited prior information.

CACHE is a public–private initiative that aims to evaluate and improve computational approaches for identifying small-molecule binders for molecular targets of pharmaceutical importance.^1^ To date, six CACHE Challenges have been organized.^17^ Each CACHE Challenge includes two rounds of predictions, thus allowing participants to leverage insights gained from the initial round for subsequent design efforts. In the CACHE Challenge #1,^15^ participants were asked to predict small molecules binding to the central cavity of the WDR domain of LRRK2. There were no known small-molecule compounds binding to the LRRK2 WDR domain at the start of the challenge. Compounds submitted by participants at the first phase and experimentally confirmed with binding assays were selected as starting points for optimization at the second phase. At this stage, participants were asked to select a new set of molecules for experimental characterization.

In this work, we developed a computational pipeline for SAR series expansion and applied it to our previously identified hit compounds for the second round of CACHE Challenge #1. Two small-molecule binders of the LRRK2 WDR domain, experimentally confirmed in the first phase of the challenge, were selected as parent molecules for the *in silico* screening of commercially available analogs. The pipeline integrated well-established computational methods, such as chemical substructure searching and molecular docking, with our recently developed workflow for AL-guided MD TI RBFE calculations and our TI simulation time optimization algorithm. Substructure searching with subsequent docking and filtering of ca. 5.5 billion commercially available small-molecule compounds allowed us to acquire a set of ca. 25K analogs of the initial hits. Leveraging the AL-RBFE workflow with optimized simulations enabled an efficient exploration of this set for analogs with improved predicted binding affinity. We identified 102 predicted hits by computing MD TI RBFE for only 672 analogs. A set of 75 predicted hits, selected based on computed MD TI RBFE, was submitted for the experimental testing. Among the 35 tested, binding assays revealed 8 hit compounds. While the binding affinity of the molecules was only in the mid-micromolar range, their confirmed binding in an orthogonal assay, the selectivity of the primary hits against an unrelated target,^12,15^ and their experimentally verified solubility and absence of aggregation at high concentration represent a solid foundation for further optimization.

Thus, our results for the first and second rounds of CACHE Challenge #1 demonstrated that the proposed approach is efficient for the *in silico* design of small-molecule binders of a challenging protein target. Starting with a known structure of the apoprotein only, we were able to first identify 5 binders with different scaffolds using deep docking in combination with MD TI ABFE calculations^12^ and then significantly expanded one of the SAR series with our AL-RBFE workflow. We believe that this approach has promising potential for streamlining and accelerating the early stages of drug discovery.

## 4 Methods

### 4.1 Database Screening and Library Preparation

The computational pipeline included the virtual screening of two sets: the pre-AL set, which contained the closest analogs of Hits 1 and 2; and the AL set, which contained general analogs of Hits 1 and 2 (see Figure 2A). Both pre-AL and AL sets were then used for AL-RBFE calculations. The individual steps of our pipeline are described below.

#### 4.1.1 Virtual Screening for Closest Analogs

##### 4.1.1.1 SMARTS Search Stage

The Enamine REAL (release as of Oct 2022) database,^10^ which contained 5.5 billion enumerated compounds, was searched for the closest analogs of Hits 1 and 2. This search was performed using SMARTS patterns (see Figure 2B, closest analogs) of Hits 1 and 2 substructures via the OpenEye Chem Toolkit.^18^ The SMARTS patterns were based on the chemical structures of the hits but with allowance for any heavy atom substitution while preserving the aromaticity and pharmacophore groups (oxamide, peptide bond, and aromatic nitrogen). This search resulted in 58 and 192 closest analogs for Hits 1 and 2, respectively. MD TI RBFEs were computed for all of the molecules from these libraries.

##### 4.1.1.2 Nearest Neighbors Search (NNS)

All Hit 1 analogs with computed negative RBFE at the time of selection were used as query molecules against a set of Hit 1 general analogs (Figure 2A). For each query molecule, 3 nearest neighbors (based on the Tanimoto distance on ECFP6-2048 bit fingerprint) were acquired from post-Template Docking library of Hit 1 analogs (*n* = 19,451) (Figure 2C), forming a list of 27 additional unique molecules for RBFE calculations.

##### 4.1.1.3 Curated Selection (CS)

Curated selection (CS, see Figure 2) was performed after RBFE calculations for the initial set of molecules selected by the SMARTS search. Ligand A, a Hit 1 analogue with the lowest computed RBFE (Figure S1), was used as the parent compound. An additional set of analogs of Ligand A was selected based on the visual inspection of general analogs of Hit 1 and MD TI RBFE. All selected molecules had a 4,5-dimethylindole ring but differed in their substituents on the 1,2,3,4-tetrahydroisoquinoline ring. This resulted in an additional set of 49 molecules for MD TI RBFE calculations.

##### 4.1.1.4 Pre-AL Set

The pre-AL set (Figure 2) included all closest analogs of Hits 1 and 2 obtained from the SMARTS search and NNS and CS stages with computed MD TI RBFE. In total, the pre-AL set included 302 molecules: 134 analogs of Hit 1 and 168 analogs of Hit 2.

#### 4.1.2 Virtual Screening for General Analogs

##### 4.1.2.1 SMARTS Search Stage

The Enamine REAL (release as of Oct 2022) database,^10^ containing ∼5.5 billion enumerated compounds, was searched for general analogs of Hits 1 and 2. The general analogue search was performed using the SMARTS pattern (see Figure 2B, general analogs) of Hits 1 and 2 substructures via the OpenEye Chem Toolkit.^18^ The SMARTS patterns were based on Murcko scaffolds of Hit 1 and Hit 2 but allowed any heavy atom substitution while preserving the aromaticity pattern. This formed libraries for the Docking Stage of 154,204 and 187,077 molecules for Hit 1 and Hit 2 general analogs, respectively.

##### 4.1.2.2 Template-Free Docking Stage

The selected ligands were docked to the minimized crystal structure of the LRRK2 WDR domain^8^ (PDB ID: 6DLO) using Glide SP.^19^ Three docked poses with the best docking scores were saved for each molecule. The protein structure prepared for docking and the parameters of Glide SP were the same as in the first phase of the CACHE Challenge #1.^12^ The docked molecules were filtered individually for Hit 1 and Hit 2 derivatives based on the docking score and the root-mean-square deviation of the indole ring-like substructure of the docked pose with respect to the indole ring of the MD representative pose (see Molecular Dynamics section 4.2.1 for details) of the corresponding hit (RMSD_indole_). The filtering included the following steps: 1) for each molecule, the best pose with minimal RMSD_indole_ with respect to the MD representative pose was selected and 2) molecules satisfying RMSD_indole_ ≤ 5 Å and Glide docking score ≤ −6 were retained. Thus, we generated libraries for further template docking of 22,428 and 26,667 molecules for Hits 1 and 2, respectively.

##### 4.1.2.3 Template Docking Stage

The MD representative structures of the LRRK2 WDR domain in complex with Hit 1 and Hit 2 were prepared for template docking using the OpenEye Make Receptor program (version 4.0.0.0). The Hit 1 and Hit 2 were set as templates, and no constraints were added. 3D conformers were generated from SMILES using OpenEye OMEGA (version 4.1.0.0). A maximum number of conformers for a single molecule of 2000 and a minimum root-mean-square deviation (RMSD) of 0.2 Å were used. Template docking was performed using an OpenEye HYBRID (version 4.0.0.0). For each molecule, the 100 best poses were stored in the output data. All other parameters were set by default.

The docked molecules were filtered based on the docking score and RMSD of the generalized Murcko scaffold of a molecule with respect to the corresponding substructure of Hit 1 or Hit 2 MD representative pose (RMSD_Murcko_). The filtering included the following steps: 1) for each molecule, one pose with minimal RMSD_Murcko_ was selected and 2) molecules satisfying RMSD_Murcko_ ≤ 4 Å, OpenEye Hybrid docking score ≤ −6, and OpenEye Hybrid docking score component for clash ≤ 0.5 were retained. This formed libraries of 19,451 and 10,070 molecules for Hit 1 and Hit 2 derivatives, respectively. The libraries were additionally filtered from duplicates based on isomeric SMILES and charged molecules. This formed the final library of 16,101 and 9,070 molecules for Hit 1 and Hit 2 derivatives, respectively, 25,171 molecules in total. This library will be termed as the AL set.

### 4.2 Alchemical Relative Binding Free Energy Calculations

#### 4.2.1 Molecular Dynamics

The docked structures of the LRRK2 WDR domain in complex with Hit 1 and Hit 2 obtained at the CACHE Challenge #1 phase 1 ^12^ were used as the initial structure for MD simulations. The protein–ligand complexes were solvated in a rectangular water box with a minimum distance between the edges of the box and the solute of 12 Å. The protein and water were parametrized using the FF14SB^20^ force field and the TIP3P^21^ model, respectively. Ligand atom parameters were obtained using GAFF2 ^22^ (version 2.11), and ligand atomic charges were derived using the AM1-BCC^23,24^ method. GPU-accelerated MD simulations were performed using the pmemd.cuda module of AMBER 20.^25–27^ The simulation protocol included the following steps: 1) 2000 steps of minimization with the gradient descent method, 2) 100 ps of heating from 1 to 298 K in the NVT ensemble, 3) 300 ps of density equilibration in the NPT ensemble, and 4) 100 ns of production simulation in NVT. Harmonic RMSD restraints were imposed on heavy atoms of the protein, ligand, and three water molecules located in the binding site during minimization and heating and were gradually removed during density equilibration. No restraints were used during the production simulations. The first 10 ns of the production MD simulation were discarded. The average structure was obtained from the last 90 ns of the simulation by averaging coordinates of ligand heavy atoms and protein C_α_ atoms. A trajectory frame with the minimum RMSD of the ligand heavy atoms and protein C_α_ atoms, with respect to the average structure, was selected as a representative structure.

#### 4.2.2 Ligand Preparation and Parameterization

Atom mapping between reference and target molecules was based on their corresponding docking poses: maximum common substructures with a maximum distance of 1.1 Å between the mapped heavy atoms were obtained via RDKit. Topologies and input coordinates for the protein–ligand complex and the solvated ligand system were generated with the FESetup^28^ v.1.2.1 software package using the generated atom mappings and MD representative structures of the protein–ligand complex for Hit 1 and Hit 2 as the input data. The ligand was parametrized with the GAFF2 force field with charges assigned via the AM1-BCC charge model. The protein was parametrized with the FF14SB force field, and the TIP3P water model was employed. The protein–ligand complex and solvated ligand box sizes employed were the same as in the calculations for the CACHE Challenge #1 phase 1.^12^

#### 4.2.3 TI Simulations

A λ-schedule following the 9-point Gaussian quadrature was employed for all simulations with softcore potentials. Each λ-window was equilibrated with 2000 minimization steps, followed by 50 ps of heating in the NVT ensemble and 300 ps of density equilibration in the NPT ensemble. On-the-fly optimization of computational resources^13^ was employed for all production simulations to minimize the computational cost of simulations. This method utilizes a short initial simulation followed by iterative automatic equilibration detection^29^ and convergence testing of the two chronological halves of the coupling potential derivative time series via the Jensen–Shannon distance, with additional simulations performed if the convergence criteria are not met. For most simulations, an initial simulation length of 2.5 ns with additional simulation lengths of 0.5 ns and a Jensen–Shannon convergence criterion of 0.1 was employed. For several simulations performed at the last AL iteration, an initial simulation length of 1.0 ns and an additional simulation length of 0.25 ns were utilized to accelerate the simulations further. When additional resources were available, multiple replicates of various transformations were performed, prioritizing those with an initially negative calculated RBFE. When multiple replicates of a transformation were performed, the ΔΔ*G* was calculated via an ensemble method in which each gradient time series of a given λ-window was individually equilibrated and decorrelated, and then all values were combined to determine overall gradient time series mean values.

### 4.3 AL Library Formation: ML-Guided Selection

Molecules for MD TI simulations were iteratively sampled from the AL set based on the recommendation of the ML model, which utilized the Active Learning approach in a similar manner to our previous work.^14^ On each iteration of the AL cycle, ML models were trained to predict ABFE via the AutoML approach. The highest performing model is then used to screen the AL set for molecules predicted to have superior ABFE. Computed RBFEs of molecules from the pre-AL set were converted to ABFEs and used to initialize the AL-RBFE workflow.

#### 4.3.1 Molecular Representations and ML Algorithms

The following featurization techniques were used: 1) RDKit molecular fingerprints (Path Fingerprints with path length 7 and binary vector length 2048, e.g., RDKFP7_2048) using RDKit; 2) Morgan fingerprints (Extended-Connectivity Fingerprints with radius 3 and binary vector length 2048, e.g., ECFP6_2048) using RDKit; 3) 3D molecular fingerprint E3FP with default parameters;^30^ 4) pharmacophore fingerprint (2D) with binary vector length 1024 (ph4fp2D_1024) using RDKit; and 5) pharmacophore fingerprint (3D) with binary vector length 1024 (ph4fp3D_1024) using RDKit.

The following ML algorithms implemented in the scikit-learn library^31^ were used: 1) Linear Regression, 2) Random Forest, and 3) Gaussian Process Regression with the Tanimoto kernel. An inner-loop 5-fold cross-validation grid search was utilized for optimal hyperparameter selection.

#### 4.3.2 Machine Learning Modeling

For each iteration of the AL cycle, ML models were trained on all molecules with available ABFEs (RBFE converted to ABFE) as the target variable. On each iteration, the ML model (combination of molecular representation and algorithm) with the highest R^2^ was selected based on leave-one-out cross-validation (LOOCV) across combinations of molecular representations and ML algorithms. After the selection of algorithm and molecular representation, the model was refitted on the entirety of the data; however, for AL iterations 1–6, ML models were trained only on Hit 1 derivatives, and the selected model was used to screen only the Hit 1 derivatives of the AL set (*n* = 16,101). For AL iteration 7, ML models were trained on Hit 1 and Hit 2 derivatives, and the selected model was used to screen the entirety of the AL set. The selection of molecules was performed greedily, harvesting the compounds with the most negative ML-predicted ABFE. Details of AL at each iteration are provided in Table S4.

### 4.4 Selection of Molecules for Experimental Validation

Molecules were selected for experimental validation according to the challenge budget (75 molecules or $10,000, whatever comes first). 70 molecules were selected solely based on the most negative computed ABFE (67 derivatives of Hit 1 and 3 derivatives of Hit 2), and the remaining 5 molecules were selected across Hit 2 derivatives with negative ABFE but biased toward chemical diversity. The selected 75 molecules were quoted by the Enamine chemical vendor. All 75 quoted molecules passed initial vendor quality control and satisfied the challenge budget.

### 4.5 Experimental Methods

#### 4.5.1 Protein Expression and Purification

DNA fragments encoding LRRK2 residues (T2124- E2527) and (T2141- E2527) were cloned into pFastBac HTA donor plasmid downstream of a His-tag or into pFBD-BirA expression vector, a derivative of Invitrogen pFastBac Dual vector for in-cell biotinylation,^32^ respectively. The resulting plasmid was transformed into DH10Bac competent E. coli (Invitrogen) to obtain recombinant viral bacmid DNA, followed by baculovirus generation for protein production in Sf9 insect cells. For in-cell biotinylation, d-biotin was added at a final concentration of 10 μg/mL during protein expression. The cells were harvested by centrifugation (2500 rpm for 10 min at 10 °C), 72–96 h postinfection with well-developed signs of infections, and 70–80% viability as previously described.^33^ Harvested cells were resuspended in 20 mM Tris-HCl, pH 7.5, 500 mM NaCl, 5 mM imidazole, and 5% glycerol; 1× protease inhibitor cocktail (100× protease inhibitor stock in 70% ethanol (0.25 mg/mL aprotinin, 0.25 mg/mL leupeptin, 0.25 mg/mL pepstatin A, and 0.25 mg/mL E-64); or Pierce Protease Inhibitor Mini Tablets, EDTA-free. The cells were lysed chemically by the addition of 1 mM PMSF, 1 mM TCEP, 0.5% NP40, and benzonase (in-house) followed by sonication at a frequency of 7.0 (5 in. on/7 in. off) for 5 min (Sonicator 3000, Misoni). The crude extract was clarified by high-speed centrifugation (60 min at 14000 rpm at 10 °C) by Beckman Coulter centrifuge. The clarified lysate was loaded onto open columns containing pre-equilibrated Ni-NTA resin (Sigma-Aldrich). The column was washed and eluted by running 20 mM Tris-HCl, pH 7.5, 500 mM NaCl, 5% glycerol, containing 5, 15, and 250 mM imidazole, respectively. The eluted proteins were then supplemented with 2 mM TCEP. The His- and Avi-tagged proteins were then further purified by size-exclusion chromatography on a Superdex200 16/600 using an KTA Pure (Cytiva) after the column was equilibrated with 50 mM Tris-HCl pH 7.5, 300 mM NaCl, and 2 mM TCEP.

For the His-tagged protein, the tag was cleaved after elution using tobacco etch virus protease (TEV) overnight while the protein was dialyzed against 20 mM Tris-HCl, pH 7.4, containing 300 mM NaCl and 2 mM TCEP. The protein was then loaded onto equilibrated Ni-NTA resin for reverse affinity to remove His-tagged TEV enzyme and the uncut His-tagged proteins. The purity and size of the cut protein were confirmed on SDS-PAGE gel and mass spectrometry, respectively, and the pure protein was concentrated and flash frozen.

#### 4.5.2 Surface Plasmon Resonance

The binding affinity of compounds was assessed by surface plasmon resonance (SPR, Biacore 8K, Cytiva Inc.) at 25 °C. Biotinylated LRRK2 (2141–2527aa—https://www.addgene.org/210899/) was captured onto the flow cells of a streptavidin-conjugated SA chip at approximately 5000 response units (RU) (according to manufacturer’s protocol). Compounds were dissolved in 100% DMSO (30 mM stock) and diluted to 10 mM before serial dilutions were prepared in 100% DMSO (a dilution factor of 0.33 was used to yield 5 concentrations). For SPR analysis, serially titrated compound was diluted 1:50 in HBS buffer (10 mM HEPES pH 7.4, 150 mM NaCl, 0.01% Tween-20) to a final concentration of 2% DMSO. Experiments were performed using the same buffer containing 2% DMSO and multicycle kinetics with a 60-s contact time and a dissociation time of 120 s at a flow rate of 40 μL/min. Kinetic curve fittings and *K*_D_ value calculations were done with a 1:1 binding model using Biacore Insight Evaluation Software (Cytiva Inc.).

#### 4.5.3 Dynamic Light Scattering

The solubility of compounds was estimated by DLS that directly measures compound aggregates and laser power in solution. Compounds were serially diluted directly from DMSO stocks and then diluted 50× into filtered 10 mM HEPES at pH 7.4, 150 mM NaCl (2% DMSO final). The resulting samples were then distributed into 384-well plates (black with a clear bottom, Corning 3540), with 20 μL in each well. The sample plate was centrifuged at 3500 rpm for 5 min before loading into DynaPro DLS Plate Reader III (Wyatt Technology) and analyzed as previously described.^34,35^

#### 4.5.4 ^19^F-NMR Spectroscopy

The binding of fluorinated compounds was assayed by observing the broadening and/or perturbation of ^19^F resonances upon addition of LRRK2 (at protein-to-compound ratios of 0.5:1 to 4:1) in PBS buffer (pH 7.4, 137 mM NaCl, 2.7 mM KCl, 10 mM Na_2_HPO_4_, 1.8 mM KH_2_PO_4_, and with 5% D_2_O). 1D-^19^F spectra were collected at 298 K on a Bruker Avance III spectrometer, operating at 600 MHz, and equipped with a QCI probe. Two to four thousand transients were collected with an acquisition period of 0.2 s, over a sweep width of 150 ppm, a relaxation delay of 1.5 s, and using 90° pulses centered at −120 ppm. The concentrations of the compounds in both reference and protein–compound mixtures were 5–10 μM. TFA (20 μM) was added as an internal standard for referencing. Prior to Fourier transformation, an exponential window function was applied (lb = 1 to 3) to the FID. All processing was performed at the workstation using the software TopSpin 3.5.

## Supplementary Material





## Data Availability

All starting structures for hit compound RBFE simulations and mutations from Hit 1 to Ligand A and from Ligand A to Ligand X are available at https://github.com/MGKurnikovaGroup/CACHE1_round_2. All simulation input files and scripts are available at https://github.com/MGKurnikovaGroup/otf_general_public.

## Notes

Number of molecules per AL iteration (Table S2), computed and measured binding affinity for optimized hits (Table S3), molecular representations for each AL cycle (Table S4), perturbation map for relative binding free energy calculations of Hit 1 (Figure S1), and full ^19^F-NMR spectra for molecules O2, O4, and O6 (Figure S2) (PDF)
Table S1: SMILES representations of all assessed molecules, characterization at primary screen, and a dose–response corresponding to Rev1 (see Table S3 for description) (XLSX)
