# Supplementary material for: Active Learning-Guided Hit Optimization for the Leucine-Rich Repeat Kinase 2 WDR Domain Based on In Silico Ligand-Binding Affinities
Source: J Chem Inf Model. 2025 May 26;65(11):5706–17. doi: 10.1021/acs.jcim.5c00588 (PMC12152950; doi:10.1021/acs.jcim.5c00588)
Supplement: Supplementary file 1 [file ci5c00588_si_001.pdf]

Supplementary Information for

Active Learning Guided Hit Optimization for the Leucine-Rich Repeat Kinase 2 WDR Domain Based on In Silico Ligand Binding Affinities

Filipp Gusev[1,2,†], Evgeny Gutkin[1,†], Francesco Gentile[3,4,†], Fuqiang Ban[5], S. Benjamin Koby[1], Fengling Li[6], Irene Chau[6], Suzanne Ackloo[6], Cheryl H. Arrowsmith[6,7], Albina Bolotokova[6], Pegah Ghiabi[6], Elisa Gibson[6], Levon Halabelian[6,8], Scott Houliston[7], Rachel J. Harding[6,8], Ashley Hutchinson[6], Peter Loppnau[6], Sumera Perveen[6], Almagul Seitova[6], Hong Zeng[6], Matthieu Schapira[6,8], ~~Olexandr Isayev[1,2,*]~~, Artem Cherkasov[5,*], Olexandr Isayev[1,2,*], Maria G. Kurnikova[1,*]

1. Department of Chemistry, Mellon College of Science, Carnegie Mellon University, Pittsburgh, PA, 15213

2. Computational Biology Department, School of Computer Science, Carnegie Mellon University, Pittsburgh, PA, 15213

3. Department of Chemistry and Biomolecular Sciences, University of Ottawa, Ottawa, ON, Canada

4. Ottawa Institute of Systems Biology, Ottawa, ON, Canada

5. Vancouver Prostate Centre, The University of British Columbia, Vancouver, BC, Canada

6. Structural Genomics Consortium, University of Toronto, Toronto, ON, M5G 1L7, Canada

7. Princess Margaret Cancer Centre, University Health Network, Toronto, Ontario, Canada

8. Department of Pharmacology & Toxicology, University of Toronto

† These authors contributed equally and share co-first authorship

* Corresponding authors: Olexandr Isayev; Artem Cherkasov; Maria G. Kurnikova

Email: olexandr@olexandrisayev.com; acherkasov@prostatecentre.com; kurnikova@cmu.edu

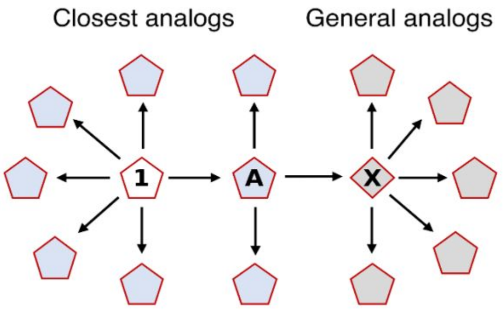

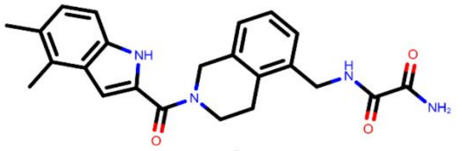

A

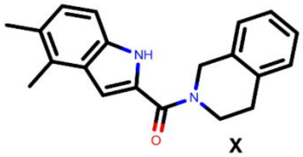

X

Figure S1. Perturbation map used for relative binding free energy calculations of Hit 1. Hit 1 is shown as a white pentagon. Structures of the Hit 1 closest analog A and intermediate compound X are provided.

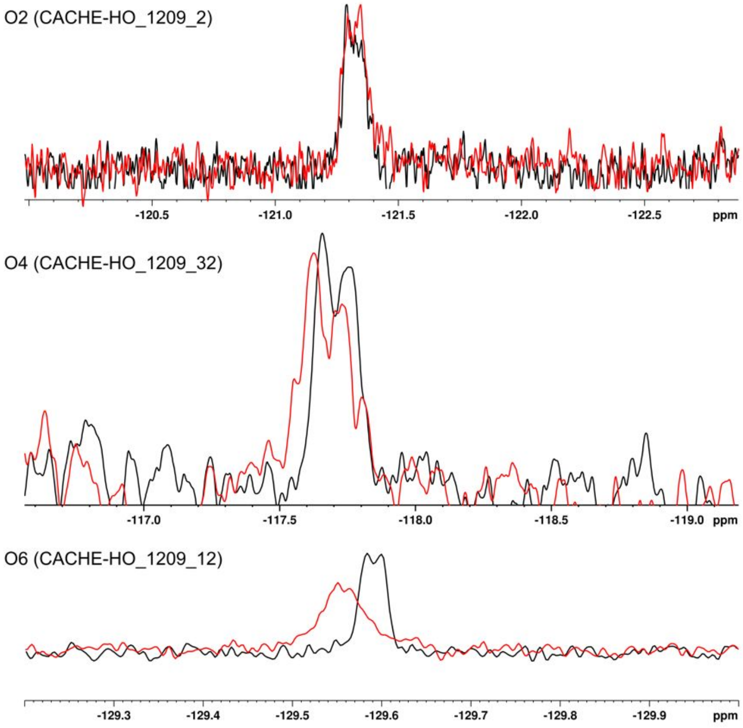

Figure S2. ^{19}F NMR spectra of fluorinated compounds O2, O4, O6 (10 µM compound with 0 [black], 20 [red] µM protein)

Table S2. Number of molecules with computed RBFEs, number of computed hits, and computed hit ratio for each iteration of AL workflow. The computed hit ratio is calculated by dividing the number of computed hits by the total number of molecules. For preAL and AL-7 iterations, data for both hits are provided. For the rest of iterations, presented data corresponds to Hit 1.

	**Hit 1 analogs/ Hit 2 analogs/ Total**		
**Iteration**	**All computed**	**Computed hits**	**Computed hit ratio, %**
preAL	134 / 168 / 302	16 / 16 /32	11.94 / 9.52 / 10.60
AL-1	89	13	14.61
AL-2	51	4	7.84
AL-3	55	15	27.27
AL-4	44	7	15.91
AL-5	49	18	36.73
AL-6	57	10	17.54
AL-7	13 /12 /25	3 / 1/ 4	23.08 / 8.33 / 16.00
**all AL**	**358 / 12 /370**	**70 / 1 / 71**	**19.55 / 8.33 / 19.19**
**Total**	**492 / 180 /672**	**86 / 17 / 103**	**17.48 / 9.44 /12.80**

Table S3. Computed and experimental binding affinities for the optimized hits. For each hit ΔG_{comp} is the computed ABFE obtained from the computed RBFE; ΔG_{exp} is the experimental binding free energies obtained from the experimental dissociation constants K_d(rev2). K_d(rev1) refers to an initial round of experimental testing, either from the first round of the competition (Hits 1 and 2) or from the second round (Hits O1-O8). Each compound was re-measured, shown as rev2. Kd (rev2) are used in the manuscript for comparisons.

Hit	Iteration	ΔG_{comp}, kcal/mol	ΔG_{exp}, kcal/mol	K_d, µM (rev2)	K_d, µM (rev1)
O1	AL-4	-5.42	-6.51	18	14
O2	AL-7	-8.60	-6.17	32	19
O3	AL-4	-5.07	-6.32	25	19
O4	AL-6	-4.88	-5.78	61	65
O5	AL-1	-5.38	-5.76	64	68
O6	AL-1	-5.43	-5.26	148	108
O7	PreAL	-5.32	-5.28	143	117
O8	AL-6	-9.71	-4.99	230	142
1	Initial hit		-6.75	12	11
2	Initial hit		-5.93	48	44

.

Table S4. AL Cycle's parameters per iteration.

AL iteration	Selected Molecular Representation	Selected for next iteration Hit 1 analogs/Hit 2 analogs/Total	Computed RBFE Hit 1 analogs/Hit 2 analogs/Total
AL-1	ECFP6_2048	119	89
AL-2	RDKFP7_2048	53	51
AL-3	RDKFP7_2048	56	55
AL-4	RDKFP7_2048	45	44
AL-5	RDKFP7_2048	52	49
AL-6	RDKFP7_2048	59	57
AL-7	ECFP6_2048	42/31/73	13/12/25
